# Safety Assessment of the Modified Lactoperoxidase System—In Vitro Studies on Human Gingival Fibroblasts

**DOI:** 10.3390/ijms24032640

**Published:** 2023-01-30

**Authors:** Marcin Magacz, Monika Papież, Dorota Kościelniak, Anna Jurczak, Karolina Kędziora, Elżbieta Pamuła, Wirginia Krzyściak

**Affiliations:** 1 Department of Medical Diagnostics, Faculty of Pharmacy, Jagiellonian University Medical College, Medyczna 9, 30-688 Kraków, Poland; 2 Doctoral School of Health and Medical Sciences, Jagiellonian University Medical College, Św. Łazarza 16, 31-008 Kraków, Poland; 3 Department of Cytobiology, Faculty of Pharmacy, Jagiellonian University Medical College, Medyczna 9, 30-688 Kraków, Poland; 4 Department of Pediatric Dentistry, Institute of Dentistry, Jagiellonian University Medical College, Montelupich 4, 31-155 Kraków, Poland; 5 Department of Biomaterials and Composites, Faculty of Materials Science and Ceramics, AGH University of Science and Technology, Al. Mickiewicza 30, 30-059 Kraków, Poland

**Keywords:** lactoperoxidase, cytotoxicity, flow cytometry, dental caries, human gingival fibroblasts, pseudohalides, cell culture

## Abstract

One strategy in caries prevention is to inhibit the formation of cariogenic biofilms. Attempts are being made to develop oral hygiene products enriched with various antimicrobial agents. One of them is lactoperoxidase—an enzyme that can oxidise (pseudo)halide ions to reactive products with antimicrobial activity. Currently, commercially available products utilise thiocyanate as a substrate; however, several alternatives that are oxidised to products with greater antimicrobial potential have been found. In this study, toxicity against human gingival fibroblasts of the lactoperoxidase system was evaluated using four different (pseudo)halide substrate systems—thiocyanate, iodide, selenocyanate, and a mixture of thiocyanate and iodide. For this purpose, cells were treated with the systems and then apoptosis, cell cycle, intracellular glutathione concentration, and mitochondrial superoxide production were assessed. The results showed that each system, after generating 250 µM of the product, inhibited cell divisions, increased apoptosis, and increased the percentage of dead cells. It was concluded that the mechanism of the observed phenomena was not related to increased superoxide production or the depletion of glutathione concentration. These findings emphasised the need for the further in vitro and in vivo toxicity investigation of the modified lactoperoxidase system to assess its safety and the possibility of use in oral hygiene products.

## 1. Introduction

In recent years, there has been an intensive search for new opportunities for the prevention and treatment of oral diseases linked to dysbiosis in the oral microbiome, which include dental caries and periodontitis [1]. Due to the relatively low effectiveness of the conventional fluoride method and the use of other synthetic antimicrobial compounds (chlorhexidine) in caries prevention, increasing antibiotic resistance, and massive costs generated for the health care system, a new approach to treating oral diseases is being sought. Latest achievements in nanotechnology (nanoparticles with antimicrobial properties or nanoparticles as drug carriers [2]), biological therapy (probiotics [3], immune response modulators [4], and cell therapy [5]), and the use of bioparticles (small molecules of natural origin [6], immunoglobulins, antimicrobial peptides, and enzymes [7]) are all promising approaches in tackling biofilm-related diseases. In the last category, one can find lactoperoxidase (LPO) which is a haemoprotein enzyme excreted by epithelial cells that is characterised by its enzymatic activity based on the oxidation of (pseudo)halides with a simultaneous reduction in hydrogen peroxide. The enzyme, along with its substrates and products, is called the lactoperoxidase system (LPO system) [8].

The activity of the LPO system is associated with the two-electron oxidation of (pseudo)halide at the expense of hydrogen peroxide [9]. In this process, the generated product can cause a disturbance in the biochemical activity of microbial cells, which is the result of the oxidation of microbial proteins (mainly their sulfhydryl and thioether groups), NAD(P)H, and small molecules associated with defence against oxidative stress [10]. In vivo, the main (pseudo)halide substrate of the LPO system is the thiocyanate ion (SCN^−^), which is the oxidated hypothiocyanite ion (OSCN^−^) [11]. However, several studies have shown that LPO is capable of oxidizing a wider range of (pseudo)halides to different reactive products (Table 1), some of which exhibit stronger antimicrobial potential than the main physiological product. However, due to the low concentration of other (pseudo)halide ions in body fluids where LPO is excreted, their physiological role as substrates for the endogenous LPO system is marginal [12,13].

The LPO system plays an important role in the defence against microorganisms within mucous membranes, mainly in the oral cavity, respiratory tract, gastrointestinal tract, and female reproductive system [14]. There are available reports suggesting that the supplementation of iodide and selenium compounds reduces the risk of infection with SARS-CoV-2, precisely via the action of the endogenous LPO system in the respiratory tract, which produces highly reactive products [17]. A significant amount of research conducted on the LPO system focusses on its role in anticariogenic defence consisting of the inhibition of metabolism or killing *Streptococcus mutans*—a causative organism used in research on dental caries [18].

Some of these studies have focused on the possibilities of using an exogenous LPO system as a way to treat and prevent caries. The exogenous administration of a LPO system consisting of an industrially isolated lactoperoxidase from cow’s milk, thiocyanate ions, and a glucose oxidase-based system to generate hydrogen peroxide has shown good results in limiting caries development by eliminating cariogenic microorganisms from the oral microbiome [19]. The current market for oral care products offers toothpastes, mouth rinses, foams, and sprays enriched with a LPO system based on the bovine-derived enzyme. Unfortunately, because the microorganisms responsible for caries development are clustered in biofilms, the action of a LPO system equipped with just a physiological substrate (SCN^−^) may yield insufficient results in terms of limiting the growth of cariogenic microorganisms. The reason for this is the relatively small oxidative potential of the product (OSCN^−^) compared with products obtained from the oxidation of nonphysiological substrates (e.g., cyanogen iodide—ICN) or compared with a product formed by a different haeme peroxidase, myeloperoxidase (HOCl) [20,21]. For this reason, a promising option appears to be the application of an exogenous LPO system enhanced with alternative (pseudo)halide substrates that are turned into products with stronger oxidative activity [11,12,15,16].

Unfortunately, even though research on the antimicrobial potential of the LPO system has been conducted for decades and recent studies have provided new discoveries regarding possibilities to boost those antimicrobial properties, there have been no comprehensive studies on the in vitro and in vivo toxicity of the LPO system toward host cells. The available literature does not unequivocally conclude if the LPO system (with SCN^−^ as the substrate) poses a threat to host cells [10,12,22,23]. However, authors have emphasised the fact that host cells are equipped with complex defence mechanisms such as the availability of intracellular and extracellular glutathione, thioredoxin, or NADH–OSCN oxidoreductase activity [24].

In light of the discoveries of new possibilities to enhance the effectiveness of the LPO system against microorganisms and the fact that, to the best knowledge of the authors, there has been no comprehensive study of the toxicity of this system against human cells, the present study was aimed to evaluate the toxicity of the products formed by the oxidation by lactoperoxidase of substrates such as SCN^−^, I^−^, SeCN^−^ against cells of human origin—human gingival fibroblasts (HGFs).

## 2. Results and Discussion

In our study, the concentrations of the individual components of the lactoperoxidase system were selected based on physiological concentrations and previously obtained results [6,14]. Furthermore, model bovine milk lactoperoxidase was used due to the fact that this enzyme is 83% similar to the human LPO found in saliva [14]. These characteristics have allowed it to be widely used in various commercially available oral hygiene products, along with other applications in food preservation [14,25]. On the one hand, the applied 5 mM concentration of (pseudo)halides, in the case of physiological substrates such as SCN^−^, reflects its salivary concentration [26], but on the other hand, this concentration guarantees an excess of (pseudo)halide substrates compared with hydrogen peroxide. The molar deficiency of hydrogen peroxide in relation to (pseudo)halide means that the amount of product is generated by the quantity of the added substrate. In the present study, the applied concentration of H_2_O_2_ of 250 μM was similar to that of previously conducted research on *Streptococcus mutans* biofilms, where this concentration applied under identical conditions was the lowest amount that triggered the antibiofilm effect [6]. All reactions were carried out at pH 7.4, which emulated the physiological state of the oral cavity in which small amounts of acids produced in the metabolic processes of both the host and the microbiota are neutralised by the buffering property of saliva.

**Figure 1:**
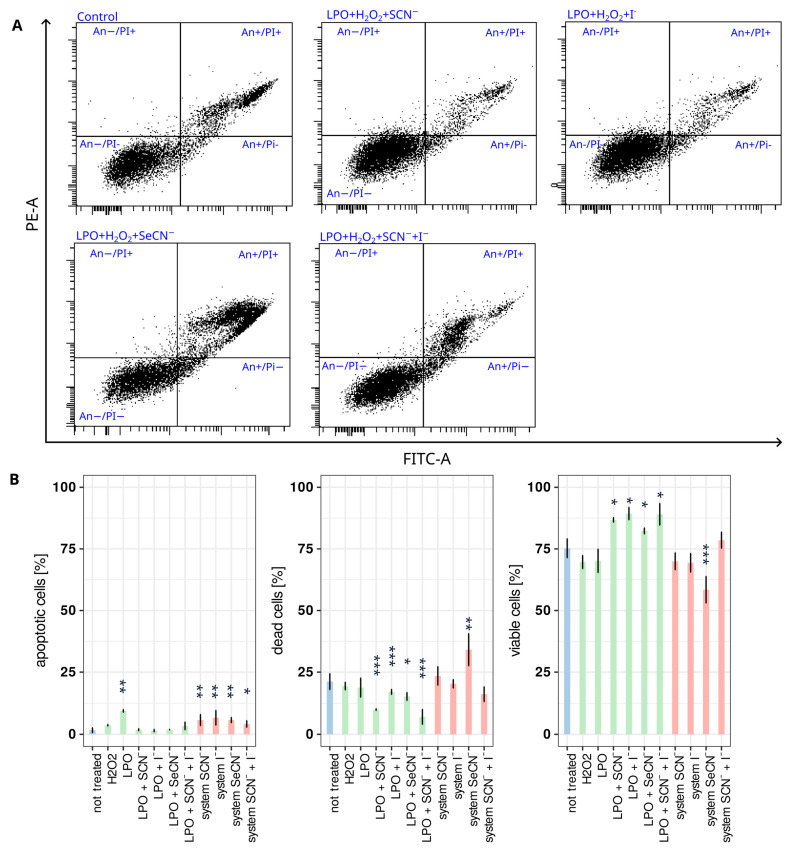
Influence of tested systems on HGF apoptosis. (**A**) Representative scattergrams of cell distribution after staining with the apoptosis assay kit. Each segment of the scattergram is labelled as: An−/Pi−—viable cells, unstained with either annexin V or propidium iodide; An+—apoptotic cells, stained with annexin V; An+/PI+—dead cells, stained with both annexin V and propidium iodide; PI+—stained with propidium iodide. (**B**) Bar plots show the percentage of apoptotic cells, dead cells, and viable cells after 1 h of treatment with tested systems. Colours indicate sample types where (i) blue denotes the control, (ii) green denotes an inactive LPO system (enzyme + (pseudo)halide), and (iii) red denotes an active LPO system. Asterisks indicate a statistically significant difference between a tested group and the control in the post hoc Tukey’s test—* *p* < 0.05; ** *p* < 0.01; *** *p* < 0.001.

Figure 1 and Table S1 show the percentage of viable, dead, and apoptotic cells among all tested groups. In comparison with the control, samples that were treated with the inactive LPO system (without the addition of hydrogen peroxide), an increase in the percentage of viable cells and a decrease in dead cells were observed. It should be noted that in the case of LPO+SeCN-, the percentage of dead cells was 28% lower than that in the control, and in samples with LPO + SCN^−^, LPO + I^−^, and LPO + SCN^−^ + I^−^, the percentage was lower by 53%, 65%, and 55%, respectively. In those samples, the percentage of apoptotic cells remained at a low level and was not significantly different from that of the control sample. This protective action may be explained by the elimination of reactive oxygen species synthetised by the LPO cells. In this reaction, toxic hydrogen peroxide is decomposed while reactive products of the LPO system are synthesised; however, its quantity is small enough that it can be efficiently eliminated by cellular defence systems such as GSH, NADPH:thiocyanate dehydrogenase, thioredoxin reductase, and proteins [27]. Hanstrom et al. [28] and Tenovuo et al. [29] described a similar protective effect in three cell lines, including HGFs. Cells that were treated with endogenously produced hydrogen peroxide (10 μM) exhibited a decrease in proliferative capacity, but the addition of a physiological LPO system (enzyme, SCN^−^) reduced the toxic effect. The results obtained in the present study are consistent with other reports and demonstrate that the LPO system plays a crucial role in defence against oxidative stress.

However, considering the short incubation time (1 h) and that the addition of exogenous H_2_O_2_ at a higher concentration than the endogenous level did not increase the percentage of dead and apoptotic cells, the protective effect of inactive LPO systems may be related to a different mechanism. HGFs are adherent cells that require detachment from the flask prior to analysis. This was made possible by using the recombinant trypsin analogue (TrypLE) as a detachment reagent. TrypLE was selected because of the lowest pro-apoptotic action among other available detachment reagents [30,31]. The relatively high percentage of dead cells observed in the control (21.18%) may have been the effect of the action of TrypLE; however, the decrease in the percentage of dead cells in the samples treated with the inactive LPO system may have been related to its protective properties during TrypLE digestion.

All tested modifications of the active LPO system showed an impact on cell death after 1 h of treatment. The strongest effect was observed for the LPO system with SeCN^−^ as the substrate, which resulted in an increase in the percentage of dead cells together with an increase in the percentage of apoptotic cells. This may suggest that this particular modification is responsible for simultaneously inducing apoptosis and necrosis. However, the LPO systems that included SCN^−^ and a combination of SCN^−^ and I^−^ weakly affected cell viability or death while statistically significantly increasing the percentage of apoptotic cells.

**Figure 2:**
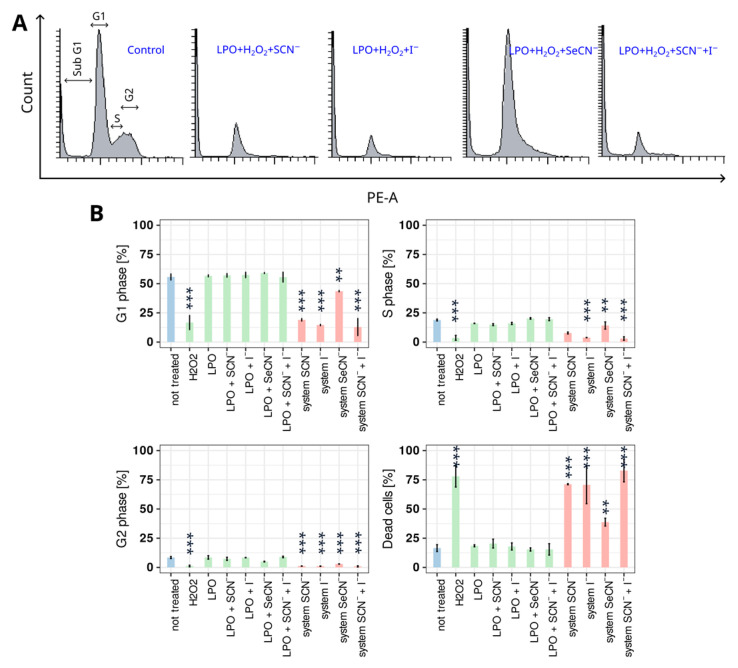
Influence of tested systems on the percentage of cells in each analysed cell cycle phase. (**A**) Representative histograms of cell cycle distribution in the control sample and samples treated with all tested active LPO systems. (**B**) Percentage (mean) of cells in each cell cycle phase. Colours indicate sample types where (i) blue denotes the control, (ii) green denotes an inactive LPO system (enzyme + (pseudo)halide), and (iii) red denotes an active LPO system. Asterisks indicate a statistically significant difference between a tested group and the control in the post hoc Tukey’s test—** *p* < 0.01; *** *p* < 0.001.

As evidenced by cell cycle analysis after 6 h of treatment, all of the tested active LPO system modifications affected cell division capacity and viability (Figure 2 and Table S2). All three LPO systems (with SCN^−^, I^−^or their mixture) inhibited HGF divisions (decreases in the cell percentage in the S and G2/M phases) and caused cell death; the increase in the dead cell percentage in the LPO system containing SeCN^−^ as a substrate was more than twice that of the control group but on average 1.9 times less than that of other samples with active modifications to the LPO system. Similarly, the inhibition of cell division was highly noticeable, but, among all the system modifications, SeCN^−^ affected those processes in the weakest way. This might have been related to the weaker redox potential of hyposelenocyanite compared with that of hypothiocyanite or reactive iodide oxidation products.

The addition of hydrogen peroxide alone caused a distinct increase in the percentage of dead cells, simultaneously lowering the number of cells in the G0/G1, S, and G2/M phases. This sample served as a positive control in the cell cycle assay due to its well-known and described apoptotic and necrotic effects on cells [32]. In samples treated with the active LPO system, the observed impact on cell cycle and viability could not be related to the toxic effect of hydrogen peroxide as that compound had been reduced to water in the reaction of the LPO system during the first minute of incubation in a separate 96-well plate before the reaction mixture was added to the cell culture. This allowed us to protect cells from the impact of hydrogen peroxide. The concentrations of substrates and enzymes were selected such that there was an excess of (pseudo)halides compared with that of hydrogen peroxide with a simultaneous high concentration of the enzyme. This approach ensured the full decomposition of hydrogen peroxide within the first seconds of the reaction such that after one minute, when the reaction mixture was transferred to the wells with cells, it was free from hydrogen peroxide.

There were no significant differences in the percentage of cells in each cell cycle in untreated samples, samples treated with only LPO, and samples treated with LPO and (pseudo)halide substrates (inactive and incomplete system). These results show that cell cycle and cell death are affected by products of the LPO system, not by enzymes or substrates on their own.

Glutathione (GSH) is one of the most important small cellular molecules responsible for protection against oxidative stress. A decrease in intracellular GSH concentration is described as one of the early signs of an apoptotic cascade. This phenomenon can be both the result of the initiation of apoptosis (the activation of the death receptor) and the cause of this process (exposure to oxidants) [33]. A prolonged decrease in the level of GSH accompanied by an increase in GSSG plays a crucial role in the initiation of apoptosis in a situation of exposure to ROS. Pias et al. demonstrated that after a significant decrease in GSH concentration, apoptosis initiation will not be triggered if the GSH concentration returns to the physiological level over a period of 60 min [33]. It was shown that due to the presence of thiol groups, GSH is a first-line defence molecule against the LPO system [34]. This may explain the decrease in HGF viability observed after the active treatment of the LPO system, related to a GSH deprivation leading to the long-term loss of redox equilibrium.

**Figure 3:**
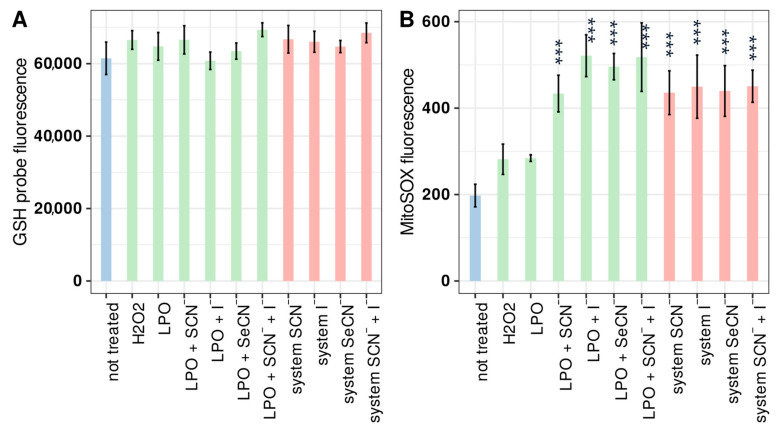
Influence of tested systems on (**A**) intracellular glutathione and (**B**) mitochondrial superoxide production in HGFs. In both cases, the bar describes the mean relative fluorescence of the cells after staining, which is proportional to the intracellular GSC concentration (**A**) and superoxide production (**B**). Colours indicate sample types where (i) blue denotes the control, (ii) green denotes an inactive LPO system (enzyme + (pseudo)halide), and (iii) red denotes an active LPO system. Asterisks indicate a statistically significant difference between a tested group and the control in the post hoc Tukey’s test—*** *p* < 0.001.

As shown in Figure 3 and Table S3, none of the LPO system modifications or substrates tested in that system had an influence on the intracellular GSH level (ANOVA *p* > 0.05). This could be explained by the cellular ability to rapidly resynthesise GSH by glutathione synthetase and glutathione reductase before irreversible changes in cells occur and apoptosis is initiated.

Reactive products of the LPO system products may deprive the activity of some key enzymes (hexokinase, 3-phosphoglyceraldehyde dehydrogenase, aldolase and glucose-6-phosphate dehydrogenase) by oxidation [14]. However, based on the obtained results, it can be assumed that glutathione synthetase and glutathione reductase are not affected by LPO products and are able to efficiently keep glutathione concentration at the physiological level. Nevertheless, further enzymological research should be conducted.

Mitochondrial superoxide analysis was conducted to assess the effect of reactive LPO system products on oxidative stress phenomena, particularly those associated with disturbances in mitochondrial electron transfer. This is related to the strong redox potential of LPO products against sulfhydryl groups, which play an important role in the activity of proteins that transport electrons and act against thioether groups in cytochrome c [35]. Mitochondrial superoxide production was significantly increased (by about 2 times in all discussed cases) (Tukey’s test *p* < 0.001) in comparison with the control in cells treated with the active LPO system and the inactive system (enzyme + (pseudo)halide substrate). Furthermore, there were no significant differences (Tukey’s test *p* < 0.05) between the active and inactive system samples (comparing samples with the same (pseudo) halide substrate), indicating that only (pseudo)halide was responsible for the observed increase in superoxide production.

The influence of iodide on oxidative stress, including mitochondrial superoxide production, was previously studied by Yao et al. using the same method (MitoSOX) [36]. Fisher rat thyroid cells treated for 2 h with 10 mM of iodide were reported to show about 20× higher MitoSOX fluorescence than the control. The research team suggested lipid and myelin peroxidation as the mechanism responsible for the observed effect. However, to the authors’ knowledge, there has been no more published research on mitochondrial superoxide production in cells treated with other (pseudo)halides and their oxidation product. Data obtained in our research demonstrate that other LPO substrates give a cell response, as was for iodide mentioned above; however, further research should be conducted to thoroughly investigate the mechanism of that action. These observations may suggest that decreases in viability, the inhibition of cell division, and intensified apoptosis are not related to mitochondrial oxidative stress, particularly the disruption of cellular respiration—more specifically disturbance of the mitochondrial electron transport chain.

Other studies suggest that the LPO system affects the cellular membrane by increasing its permeability. In a simple study model, Odajima et al. [22] showed that the LPO system containing SCN and I- as substrates induced haemolysis in human, swine and equine erythrocytes. On the one hand, such results may suggest an important role of the LPO system in increasing the permeability of human cell membranes. On the other hand, it is important to note that erythrocytes are particularly susceptible to changes in chemical and physical condition, mainly due to their characteristic structure and biochemistry. Similar conclusions were drawn by Ihalin et al., who examined the effect of an analogue of a LPO system, horseradish peroxidase (HRP), with iodide as the substrate [10]. The team determined that the LPO system was found to inhibit cell metabolism in HGFs, skin fibroblasts, and keratinocytes using the Alamar Blue assay. Furthermore, HGFs were found to be more susceptible to increased cell membrane permeability after HRP/I^−^ system treatment compared with the other two cell types [10]. Considering both the results of the present study and the results reported in the literature, it is possible to define the main cytotoxic mechanisms of the action of the LPO system against HGFs as: (i) increasing cell membrane permeability, (ii) affecting intracellular components such as proteins involved in biochemical processes, and (iii) affecting nucleotides engaged in redox reactions. The obtained results do not suggest that the studied LPO systems are responsible for increasing oxidative stress in GSH depletion or disturbing the mitochondrial electron transport chain in treated cells.

It should be emphasised that the observed in vitro toxicity effects of (pseudo)halide oxidation products (generated by LPO) do not necessarily translate into in vivo toxicity. A literature search in PubMed and Embase did not reveal any studies on the subject of the application of the safety of the LPO system in vivo, more specifically in the oral cavity. One article focused on studies of dermal toxicity in rabbits using the Buehler method, concluding that no skin sensitisation was observed after treatment with reactive products of iodide oxidation and horseradish peroxidase [23]. It should also be noted that the oral cavity environment is rich in various protective systems that protect host cells from the toxic effects of LPO activity. These mechanisms include a consistent flow of saliva that constantly removes products from the LPO system of both endogenous and exogenous origin. Furthermore, saliva itself contains antioxidants, particularly glutathione, which contains the thiol group and is one of the main targets of the reactive products of the LPO system [37]. Other less specific targets present in saliva are proteins, peptides, and amino acids [38]. Various kinds of immunomodulators such as leukocytes, mesenchymal stem cells [5], and their products—cytokines and intracellular exosomes [4]—play important roles in the regulation of in vivo inflammation processes. In future applications of modified lactoperoxidase systems in oral hygiene products, the recommended exposure time is approximately 2 min for toothpaste and 1 min for mouth rinse, which is substantially shorter than the exposure applied in an assay [39]. After that, rinsing the mouth with water and subsequent saliva flow would ensure the elimination of the LPO system products.

It should be noted that following the acidification of the oral cavity related to a mono- and disaccharide-rich diet is an increase in the undissociated form of reactive products of the LPO system in relation to the dissociated form. Undissociated hypo(pseudo)halides penetrate cell membranes more easily, which accounts for their stronger toxic effect [11]. The present study was conducted to ensure stable conditions for in vitro cell cultures where all reactions were run in PBS (pH = 7.4), which in turn caused a shift of the dissociation equilibrium to an advantageous dissociated form of the LPO system.

One of the limitations of the presented study is that it only assessed short-term effects related to oxidation stress and cell death with the application of one previously selected concentration of LPO system products. The obtained results should be considered a starting point for future planned studies that, above all, should be directed at the evaluation of the long-term effects of cell exposure to LPO system products, mainly genotoxicity, mutagenicity, and general impact on nucleic acids. This study, similarly to other in vitro research, did not allow us to fully evaluate the toxicological safety of modified LPO systems due to a multitude of factors that can either exhibit protective properties (as described above) or enhance the toxic action of LPO. Accordingly, further research on models applying the mentioned conditions that can occur in vivo, including the presence of multiple protective systems, must be conducted. Reaching this goal might be accomplished by conducting studies in vivo, firstly on animal models, which should include the morphological and histological evaluation of tissues, as well as the evaluation of genotoxicity and potential biochemical changes after exposure.

Another limitation of the presented research is one particular simplification applied in to practically trigger the lactoperoxidase system reaction. Here, the substrate for LPO, which was hydrogen peroxide, was delivered in substantia in one portion that, in turn, provided the practically instantaneous formation of final amount of product. In this regard, it is important to keep in mind the fact that in future practical applications (oral hygiene products and antimicrobial products), this substrate would not be directly added to the formula due to the need to activate the system not during the preparation of the formula but only at the targeted site due to the low stability of reactive LPO products. In this case, however, hydrogen peroxide would be generated by an additional enzyme (e.g., glucose oxidase) that would release this substrate in a controlled manner while simultaneously regulating the synthesis rate of LPO products. This would contribute to the lowering of the temporary concentration of LPO products and prolonging the duration of LPO system action. A similar process takes place for an endogenous LPO system where hydrogen peroxide is continuously provided in small amounts as a result of dual oxidase 2 (DUOX2) [40] activity in host cells, and its synthesis carried out by oral microbiome [14].

A significant practical application of the obtained data on LPO system toxicity against host cells is the possibility of more focused future research on LPO system modifications and their impact on biofilms by our research team and others. The above-mentioned microbiological and toxicological studies should be mutually oriented and complement each other in order to achieve the goal of selecting such systems that are characterised by strong antibiofilm activity and do not show toxicity towards host cells. The achievement of this goal is the first step to the implementation of the created LPO systems and practical clinical application in the form of preparations (toothpastes, mouthwashes, and lozenges combined with polyols) used in the prevention and treatment of oral diseases associated with dysbiosis, such as dental caries and periodontitis.

## 3. Materials and Methods

### 3.1. Lactoperoxidase System

Four modifications of the lactoperoxidase system were tested. Each modification consisted of using a different (pseudo)halide substrate for LPO (as listed in Table 1). In each experiment, the concentration of the given (pseudo)halide was 5 mM. All of the (pseudo)halide substrates were used as potassium salts (KSCN, KI and KSeCN) with a minimum purity of 99% (Merck, Schnelldorf, Germany).

Bovine lactoperoxidase (Merck, Schnelldorf, Germany) was used as a substitute for human LPO because of its very high similarity in structure and chemical properties. Before each experiment, the freeze-dried portion of the enzyme was dissolved in PBS and the LPO concentration was determined with UV–VIS spectroscopy (ε_412nm_ = 112,000) (Thermofisher Multiskan Sky, Waltham, MA, USA). In all experiments, a final LPO concentration of 50 nM was used.

Hydrogen peroxide (ACS reagent, Merck, Schnelldorf, Germany) was used as a second substrate for LPO. The hydrogen peroxide concentration was determined each time right before the start of an experiment using direct UV–VIS spectroscopy (ε_240nm_ = 43.6) (Thermofisher Multiskan Sky, Waltham, MA, USA). In all experiments, a H_2_O_2_ concentration of 250 μM was used.

### 3.2. Cell Culture Conditions

An adherent cell line of primary human gingival fibroblasts (ATCC PCS-201-018) was purchased from the American Type Culture Collection (ATCC, Manassas, VA, USA). The cells were cultured in a fibroblasts basal medium (FBM) (ATCC PCS-201-030) supplemented with Fibroblast Growth Kit-Serum-free ATCC PCS-201-040 (HSA, linoleic acid, lecithin, L-glutamine, rhFGF, rhEGF, rhinsulin, hydrocortisone, and ascorbic acid). Before proper experiments, cells were passaged twice, counted in a haemocytometer, divided into portions, added to 2 mL cryotubes, and frozen in liquid nitrogen until use. Cells were cultured in a Thermo Scientific Nunc EasYFlask (75 cm^2^ Delta Surface flasks). Before each experiment, the cells were thawed and divided so that each flask contained 300,000 cells. Next, cells were grown to reach >80% of confluence, with the medium changed every two days. The cells were passaged into new flasks (300,000 cells/flask) until reaching the third passage. After each passage, the cell monolayer was washed with PBS and trypsinised using a TrypLE dissociation reagent (ThermoFisher, Watlham, MA, USA) to obtain a cell suspension that was immediately diluted with FBM, centrifuged, resuspended in fresh FBM, and counted.

### 3.3. General Course of Experiments on HGFs

Before each experiment, a single cell suspension was prepared via the trypsinization (TrypLE) of a monolayer cell culture followed by resuspension in fresh FBM and then counted. A portion of 100,000 cells was added to a 24-well plate (300,000 in a 6-well plate for cell cycle assay) in 1 mL (3 mL for cell cycle assay) of supplemented FBM. The cells in the plates were incubated for 24 h at 37 °C and 5% CO_2_ to achieve the full adhesion and confluence of the fibroblasts. After incubation, enzymes and substrates were prepared in a separate 96-well plate, each well holding 200 μL of the mixture. After 1 min of the propagation of the enzymatic reaction (which allowed the reaction to completely run, consuming all available substrates), the mixtures were transferred to cell cultures and incubated for 1 h (six hours in the case of the cell cycle assay). Subsequently, the cells were washed with PBS, detached from the bottom of the well using TrypLE, washed with PBS, centrifuged, resuspended in FACS tubes, and used for the flow cytometry staining procedure. The control sample of all experiments was obtained by adding of 200 μL of PBS to HGFs. All samples were tested in at least triplicate.

### 3.4. Apoptosis Assay

To determine apoptosis after treatment, HGF cells were stained using an apoptosis flow cytometry kit with annexin V conjugated with Alexa Fluor 488 (ThermoFisher, Waltham, MA, USA). Cells were resuspended in HEPES with a Ca^2+^ binding buffer in FACS tubes (the cell count of the sample was 10^5^) and stained with annexin V–Alexa Fluor 488 V (5 μL) and propidium iodide (1 μL). After 15 min of incubation at 37 °C, 400 μL of an annexin binding buffer was added. Subsequently, the cells were analysed by flow cytometry with the acquisition of 20,000 single cells.

### 3.5. Cell Cycle Assay

Cell cycle analysis was performed using the propidium iodide staining method with flow cytometry detection [41,42]. Treated and washed cells (10^5^) were fixed and permeabilised via suspension in 70% freezing cold ethanol in deionised water. After 24 h of incubation at −20 °C, cells were triple-washed with PBS and ultimately resuspended in 100 μL of PBS. Staining was carried out by adding 200 μL of a staining solution (3 µg/mL of bovine pancreatic RNase DNase-free (Merck, Schnelldorf, Germany) and 50 µg/mL of propidium iodide in PBS (Merck, Schnelldorf, Germany)) to the cell pellet. After incubation at 37 °C for 15 min, cells were subsequently analysed by flow cytometry.

### 3.6. Mitochondrial Superoxide Production Assay

Mitochondrial ROS formation after treatment was assessed using the MitoSOX kit (ThermoFisher, Watlham, MA, USA) modified as described by Kauffman et al. [43]. First, 10^5^ cells were resuspended in 500 μL of PBS, and then 1 μL of a 2.5 μM MitoSOX dye solution in DMSO was added. After 20 min of incubation at 37 °C, cells were centrifuged and resuspended in 500 μL of fresh PBS. Subsequently, the cells were analysed by flow cytometry.

### 3.7. Intracellular GSH Assay

Intracellular glutathione in cells treated with the tested systems was determined by flow cytometry using a fluorescence staining kit (Abcam, Cambridge, UK). Cells were suspended in 500 μL of an assay buffer, and 2.5 μL of Thiol Green Dye was added. After 20 min of incubation at 37 °C, cells were centrifuged and resuspended in 500 μL of fresh PBS. Subsequently, the cells were analysed by flow cytometry.

### 3.8. Flow Cytometry Analysis

Flow cytometry cell analysis (Becton Dickinson Biosciences Immunocytometry Systems, San Jose, CA, USA) was performed until the acquisition of 20,000 single cells at the target gate. Fluorescence was excited with a 488 nm blue laser (530/30 BP filter and 575/26 BP filter were used). Doublets and debris were excluded using gating on width versus area of the forward scatter (FSC) and side scatter (SSC). Data were acquired and analysed with the BD FACSDiva software (v9.0, Becton Dickinson Biosciences Immunocytometry Systems, San Jose, CA, USA) [44].

### 3.9. Statistical Analysis

The R Studio software (v2022.07.1+554, Posit, Boston, MA, USA) with an R environment (V4.2, R Core Team, Worldwide) was utilised for data analysis with the ggplot2 package for plot preparation. A one-way analysis of variance (ANOVA) with the post hoc Tukey’s test was used to calculate the statistical significance between groups before assessing the homogeneity of variances with the Levene’s test. All analyses were performed at a 95% confidence level.

## 4. Conclusions

The results obtained in the presented study demonstrated that the lactoperoxidase system modulated with four chosen (pseudo)halide substrates may exhibit a cytotoxic effect on human gingival fibroblasts. However, based solely on in vitro studies, each particular modification of the LPO system cannot be explicitly concluded as safe or dangerous to host cells without further investigation in in vivo animal models.

## Figures and Tables

**Table 1:** Substrates and products of the lactoperoxidase (pseudo)halogenation cycle that are the subject of the present study.

Substrate	Product	Source
Thiocyanate (SCN^−^)	Hypothiocyanite (OSCN^−^)	[14]
Selenocyanate (SeCN^−^)	Hyposelenocyanite (OSeCN^−^)	[12]
Iodide (I^−^)	Reactive iodide species (HOI, I_2_OH, I_2_^−^, I_3_^−^, I_5_^−^, I_6_^−^, I_2_)	[11]
Mixture of I^−^ and SCN^−^	Cyanogen iodide (ICN), reactive iodide species, OSCN^−^	[15,16]

## Data Availability

Not applicable.

## Supplementary Materials

The supporting information can be downloaded at: https://www.mdpi.com/article/10.3390/ijms24032640/s1.
