# Supplementary material for: Safety Assessment of the Modified Lactoperoxidase System—In Vitro Studies on Human Gingival Fibroblasts
Source: Int J Mol Sci. 2023 Jan 30;24(3):2640. doi: 10.3390/ijms24032640 (PMC9916481; doi:10.3390/ijms24032640)
Supplement: Supplementary file 1 [file ijms-24-02640-s001.zip › ijms-2132842-supplementary.pdf]

International Journal of
Molecular Sciences

MDPI

Supplementary Materials

Table S1. Percentage ± standard deviation of viable, dead and apoptotic cells after 1 h of treatment with different modification of active LPO system and inactive LPO system (without the addition of hydrogen peroxide). Asterisks indicate statistically significant difference between a tested group and control in post hoc Tukey's test - *p<0.05; **p<0.01; ***p<0.001.

HGF Treatment	Viable Cells [%]	Dead Cells [%]	Apoptotic Cells [%]
Control	75.27 ± 3.83	21.18 ± 3.21	3.22 ± 0.96
H_2O_2	69.67 ± 2.67	19.37 ± 1.52	3.67 ± 0.32
LPO	70.17 ± 4.76	18.73 ± 3.85	9.43 ± 0.56**
LPO + SCN^-	86.77 ± 0.93*	9.97 ± 0.35***	1.87 ± 0.42
LPO + I^-	89.57 ± 5.44*	7.4 ± 4.85**	2.40 ± 0.72
LPO + $SeCN^-$	82.30 ± 1.18*	15.20 ± 1.56*	1.90 ± 0.10
LPO + SCN^- + I^-	86.76 ± 1.48*	9.43 ± 1.03***	2.00 ± 1.21
Active system			
LPO + SCN^- + H_2O_2	69.97 ± 3.44	23.47 ± 3.77	5.73 ± 2.24**
LPO + I^- + H_2O_2	69.36 ± 3.80	20.26 ± 1.71	6.67 ± 2.83**
LPO + $SeCN^-$+ H_2O_2	58.43 ± 5.39***	34.10 ± 6.51**	5.73 ± 1.05**
LPO + SCN^- + I^-+ H_2O_2	78.53 ± 3.27	16.10 ± 2.95	4.17 ± 1.32*

Table S2. Percentage ± standard deviation of cells in G0/G1, S, G2 phase and dead cells after 6h of treatment with different modification of active LPO system and inactive LPO system (without the addition of hydrogen peroxide). Asterisks indicate statistically significant difference between a tested group and control in post hoc Tukey's test - *p<0.05; **p<0.01; ***p<0.001.

HGF Treatment	Cell Cycle Phase			
	G0/G1 [%]	S [%]	G2 [%]	Dead Cells [%]
Control	55.7 ± 2.6	18.9 ± 0.8	8.5 ± 0.9	16.5 ± 2.9
H_2O_2	16.7 ± 6.0***	3.6 ± 2.1***	1.2 ± 0.7***	78.0 ± 9.1***
LPO	56.7 ± 0.8	16.0 ± 0.8	8.6 ± 0.9	18.5 ± 0.8
LPO + SCN^-	57.1 ± 1.6	14.9 ± 0.8	7.4 ± 1.3	20.4 ± 3.8
LPO + I^-	57.4 ± 2.3	16.0 ± 0.9	8.4 ± 0.1	18.0 ± 3.0
LPO + $SeCN^-$	59.1 ± 0.4	20.2 ± 0.6	5 ± 0.3	15.4 ± 1.3
LPO + SCN^- + I^-	55.6 ± 4.2	19.6 ± 1.2	8.9 ± 0.6	15.4 ± 4.8
**Active systems**				
LPO + SCN^- + H_2O_2	19.0 ± 1.0***	7.7 ± 0.8***	1.13 ± 0.1***	71.2 ± 0.5***
LPO + I^- + H_2O_2	14.6 ± 0.6***	3.8 ± 0.1***	1.0 ± 0.1***	70.7 ± 16.1***
LPO + $SeCN^-$+ H_2O_2	43.6 ± 0.6**	14.2 ± 0.1**	0.8 ± 0.2***	38.8 ± 3.3*
LPO + SCN^- + I^-+ H_2O_2	12.9 ± 7.3***	3.0 ± 1.5***	0.9 ± 0.5***	82.9 ± 9.6**

Table S3. Absolute fluorescence intensity ± standard deviation of probes detecting cellular GSH and mitochondrial superoxide after 1 h treatment of different modifications of active LPO system and inactive LPO system. Asterisks indicate statistically significant differences between a tested group and control in Tukey's test – *p<0.05; **p<0.01; ***p<0.001.

HGF Treatment	Mean GSH Fluorescence	Mean MitoSOX Fluorescence
Control	61478 ± 4473	197.7 ± 26.2
H_2O_2	66539 ± 2573	281.7 ± 35.1
LPO	64774 ± 3829	284.3 ± 7.6
LPO + SCN^-	66570 ± 3900	433.7 ± 42.5***

LPO + I^-	60797 ± 2407	521.3 ± 48.4***
LPO + $SeCN^-$	63474 ± 2238	496.0 ± 30.4***
LPO + SCN^- + I^-	69376 ± 1897	518.0 ± 79.4***
**Active systems**		
LPO + SCN^- + H_2O_2	66734 ± 3807	435.6 ± 50.6***
LPO + I^- + H_2O_2	66049 ± 2905	449.7 ± 73.0***
LPO + $SeCN^-$+ H_2O_2	64717 ± 1677	439.7 ± 42.5***
LPO + SCN^- + I^-+ H_2O_2	68511 ± 2727	450.7 ± 37.2***
